# Repurposing phenformin for the targeting of glioma stem cells and the treatment of glioblastoma

**DOI:** 10.18632/oncotarget.10919

**Published:** 2016-07-29

**Authors:** Wei Jiang, Susan Finniss, Simona Cazacu, Cunli Xiang, Ziv Brodie, Tom Mikkelsen, Laila Poisson, David B. Shackelford, Chaya Brodie

**Affiliations:** ^1^ Davidson Laboratory of Cell Signaling and Tumorigenesis, Hermelin Brain Tumor Center, Department of Neurosurgery, Henry Ford Hospital, Detroit, MI, USA; ^2^ Department of Public Health Sciences, Henry Ford Hospital, Detroit, MI, USA; ^3^ Department of Pulmonary and Critical Care Medicine, UCLA David Geffen School of Medicine Los Angeles, CA, USA; ^4^ Everard and Mina Goodman Faculty of Life Sciences, Bar-Ilan University, Ramat-Gan, Israel

**Keywords:** glioma stem cells, phenformin, non-cording RNAs, HMGA2, dichloroacetate

## Abstract

Glioblastoma (GBM) is the most aggressive primary brain tumor with poor prognosis. Here, we studied the effects of phenformin, a mitochondrial complex I inhibitor and more potent chemical analog of the diabetes drug metformin on the inhibition of cell growth and induction of apoptosis of glioma stem cells (GSCs) using both *in vitro* and *in vivo* models. Phenformin inhibited the self-renewal of GSCs, decreased the expression of stemness and mesenchymal markers and increased the expression of miR-124, 137 and let-7. Silencing of let-7 abrogated phenformin effects on the self-renewal of GSCs via a pathway associated with inhibition of H19 and HMGA2 expression. Moreover, we demonstrate that phenformin inhibited tumor growth and prolonged the overall survival of mice orthotopically transplanted with GSCs. Combined treatments of phenformin and temozolomide exerted an increased antitumor effect on GSCs *in vitro* and *in vivo*. In addition, dichloroacetate, an inhibitor of the glycolysis enzyme pyruvate dehydrogenase kinase, that decreases lactic acidosis induced by biguanides, enhanced phenformin effects on the induction of cell death in GSCs and prolonged the survival of xenograft-bearing mice. Our results demonstrate for the first time that phenformin targets GSCs and can be efficiently combined with current therapies for GBM treatment and GSC eradication.

## INTRODUCTION

Glioblastoma (GBM) is the most common and aggressive astrocytic tumor and is characterized by increased proliferation, invasion into the surrounding normal tissue, robust angiogenesis and resistance to conventional therapies [1]. The prognosis for patients with GBM remains extremely poor and has not changed significantly during the last decades. GBM contain a small subpopulation of cancer stem cells (glioma stem cells, GSCs) [2] that are characterized by the ability to self-renew, exhibit multi-lineage differentiation potential and to generate xenografts that recapitulate the parental tumors [3]. Furthermore, GSCs are resistant to conventional therapies as compared with differentiated tumor cells, and therefore remain at the tumor site following resection, which eventually leads to tumor recurrence [3, 4]. Therefore, identifying therapeutic approaches to selectively target GSCs is of great importance for the treatment of GBM and for improving patient prognosis.

Metformin is a biguanide drug that has been used clinically for the treatment of type II diabetes for decades. Epidemiological studies have suggested an anti-cancer impact of metformin in diabetic patients [5]. Additional studies have shown that metformin can inhibit cancer cell proliferation and induce cell cycle arrest in multiple cancer types, including glioma [6–10]. Importantly, metformin inhibits the growth of cancer stem cells (including GSCs), induces tumor regression and prolongs tumor remission in xenograft models [8, 11]. These findings suggest that metformin could be a promising drug alone and in combination with other treatments to target GSCs.

Phenformin, an analog of metformin, exhibits a greater anti-tumor activity [12] and potency in leukemia, lung, breast, colon, melanoma and prostate cancers [13–16]; however, no studies regarding the effect of phenformin on GBM and GSCs have been reported. The primary target of biguanides in the cells is the mitochondria, where these drugs transiently inhibit complex I of the mitochondrial electron transport chain, which results in decreased ATP production and increased AMP levels [17]. These processes in turn activate 5′-AMP-activated protein kinase (AMPK), an energy sensor and a master coordinator of integrated signaling networks consisting of metabolic and growth pathways [5]. AMPK activation following biguanide-induced metabolic stress has been shown to inhibit mammalian target of rapamycin complex 1 (mTORC1) leading to reduced growth in tumor cells [18–20]. In addition to the known effects of metformin and phenformin on cell metabolic pathways, there are additional processes that are implicated in their antitumor effects such as inhibition of the NF-κB and STAT3 pathways [21–24]. Metformin also targets cancer cells via modulation of small noncoding RNAs (miRNAs) that inhibit gene expression at the posttranslational level. Several miRNAs, such as miR-33a, miR-26a, miR-193, miR-221/222 and let-7, are regulated by metformin in breast, pancreatic, and lung cancer cells [25–29]. In contrast, no studies on the regulation of miRNAs by phenformin were reported so far.

Here, we studied the effects of phenformin on GSC stemness and apoptosis and analyzed the molecular mechanisms involved in its effects. In addition, we analyzed the effects of phenformin alone and in combination with temozolomide (TMZ) and dichloroacetate (DCA) on GSCs *in vitro* and on the growth of GSC-derived xenografts and animal survival.

## RESULTS

### Phenformin inhibits GSC self-renewal and stemness

**Figure 1:**
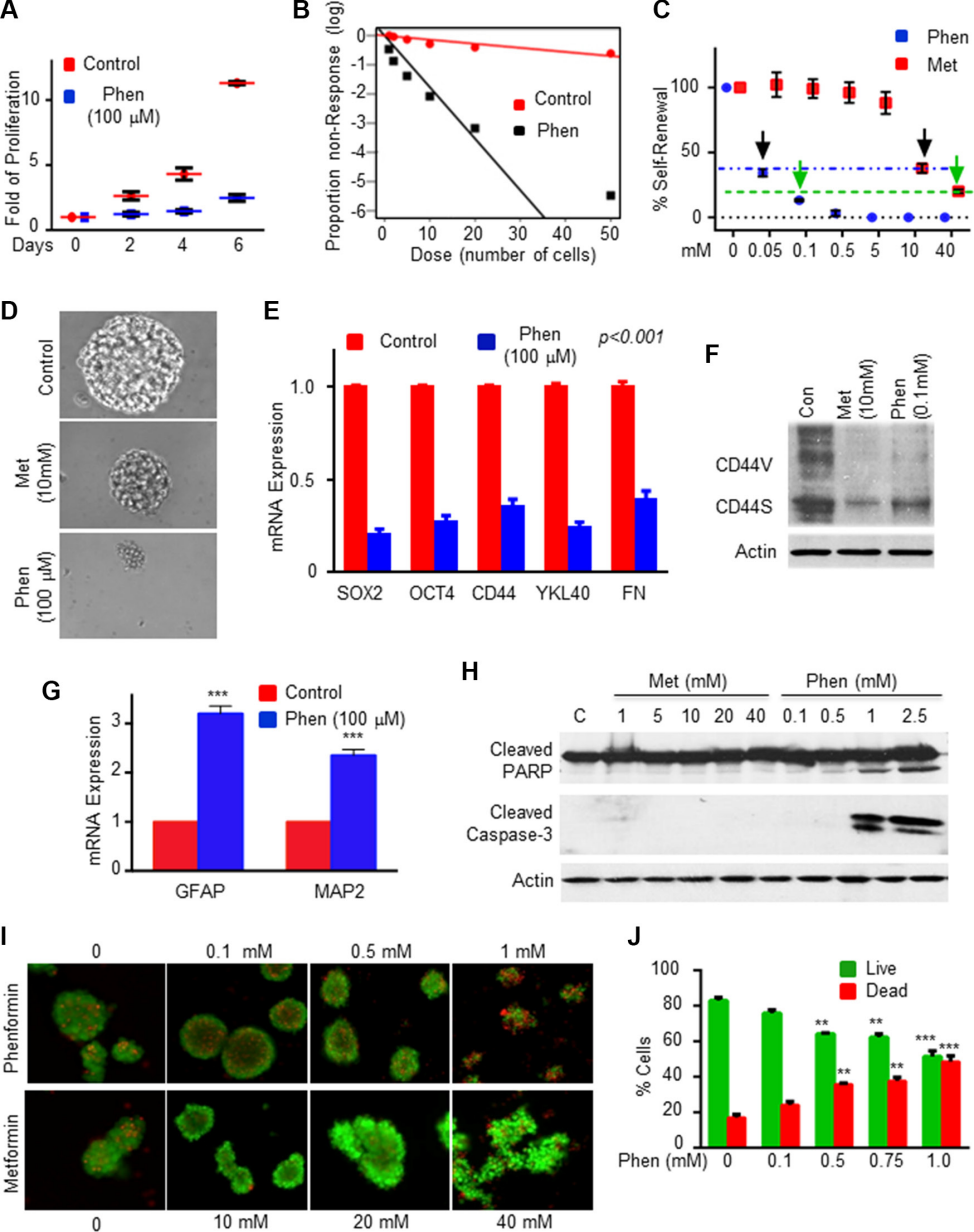
Phenformin inhibits GSC self-renewal and induces GSC apoptosis (**A**) HF2354 and HF2414 GSCs were treated with 100 μM phenformin and cell proliferation was determined at different time points in culture. (**B**) *In vitro* extreme limiting dilution assay (ELDA) demonstrated that phenformin treatment decreased the frequency of neurosphere formation (HF2354 GSCs). (**C**) Self-renewal analysis was performed with three different GSCs (HF2587, HF2414 and HF2354). Control or treated-GSCs were plated at 10 cells/well in 96-well plates and the number of neurospheres per well was quantified after 10 days. *p* < 0.0001. (**D**) Representative pictures of neurosphere size after 2 weeks of treatment (HF2354) are presented. (**E**) The expression of stemness and mesenchymal markers in HF2355 GSCs that were treated with phenformin (100 μM) for 3 days was determined using qPCR and for CD44 (**F**) using also Western blot analysis. (**G**) Expression of GFAP and MAP2 mRNA in phenformin (100 μM, 3 days) treated GSCs (HF2355). (**H**) Western blot analysis of cleaved PARP and caspase-3 in GSCs after 24 hours treatment. (**I**) GSCs were treated with various concentrations of phenformin or metformin for 24 hr and cell death was determined using the live (green)/dead (red) assay. (**J**) Quantification of the dead and live cells is presented. E–J represent the results of at least three different experiments/samples that gave similar results. For statistical analysis, *p < 0.05, **p < 0.01, ***p < 0.001, ****p < 0.0001.

Cancer stem cells are resistant to chemotherapy and radiation therapy and are implicated in tumor infiltration and recurrence. Previous studies suggested that metformin selectively targeted cancer stem cell growth in breast, lung, melanoma and glioma tumors [8, 10, 30–35]. However, the effects of phenformin on GSCs are not yet described. To examine whether phenformin can target GSCs, we employed neurosphere cultures that were generated from three individual GBM primary tumors. The GSCs were maintained as spheroids in serum-free medium containing FGF and EGF and their self-renewal, differentiation and tumorigenic abilities were validated as previously reported [36–40]. We examined the effects of phenformin on the self-renewal and stemness of these cells and included metformin for comparison in some of these studies. We found that treatment of the HF2414 GSCs with phenformin (100 μM) significantly decreased the proliferation of the GSCs (Figure 1A). In addition, phenformin also inhibited the frequency of sphere formation (Figure 1B) and the self-renewal of these cells (Figure 1C). Dose-response analysis indicated that the inhibitory effect of phenformin on the self-renewal of the cells was observed already at a concentration of 50 μM, whereas the inhibitory effects of metformin were first observed at a concentration of 10 mM (Figure 1C). In addition, GSCs were more sensitive to phenformin treatment even though phenformin concentration was already 400-fold lower than that of metformin (comparison of the self-renewal level is indicated by the green arrows in Figure 1C). Similar results were obtained with additional GSCs (Supplementary Figure S1A). Moreover, the average sphere size of the phenformin-treated GSCs was much smaller than that of untreated spheroids or those treated with metformin (Figures 1D and Supplementary S1B).

To further confirm that phenformin can affect GSC stemness, we analyzed the expression of the stemness markers OCT4, SOX2 and CD44 in the treated cells and found that phenformin (100 μM) inhibited the expression of these markers (Figure S1E, 1F, Supplementary Figure S1C–S1E), whereas it increased the expression of the neural markers, GFAP and MAP2 (Figures 1G and Supplementary Figure S1C). In addition, we found that phenformin decreased the expression of YKL40 and fibronectin, which are associated with the mesenchymal transformation of GSCs (Figure 1E and Figure S1C). Similar effects on stemness markers were obtained with metformin, however, these effects were observed only at a concentration of 20 mM (Supplementary Figure S1D and S1E).

Phenformin at concentrations up to 500 μM did not induce significant GSC death (Figures 1H–1I), but cell apoptosis was induced by phenformin at concentrations higher than 1.0 mM already after 24 hr of treatment as documented by the increases in cleaved caspase 3 and PARP expression (Figure 1H) and using the live/dead assay (Figures 1I, 1J). In contrast, metformin induced only a small degree of cell death after 24 hours at a concentration of 40 mM that was observed using the live/dead assay (Figure 1I) but not by analyzing cleaved caspase-3 (Figure 1H). In addition, we also found that the effect of phenformin on GSCs was much more potent than its effect on glioma cell lines (Supplementary Figures S1F, S1G). Phenformin barely induced caspase-3 and PARP cleavage in differentiated glioma cells when its concentration was 1 mM.

### Phenformin regulates the expression of non-coding RNAs and inhibits the self-renewal of GSCs via the H19/let-7/HMGA2 pathway

**Figure 2:**
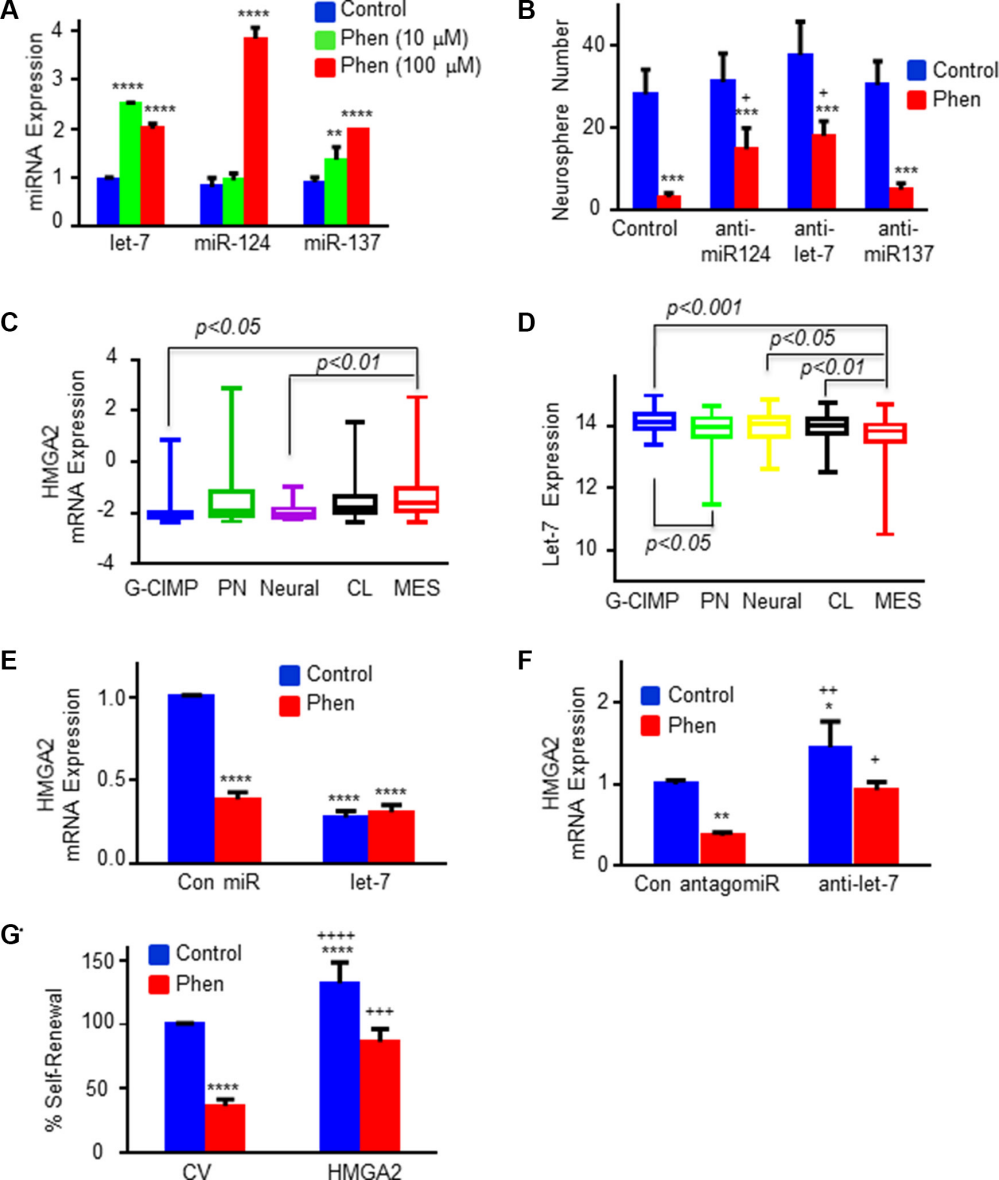
Phenformin regulates the expression of specific miRNAs and HMGA2 in GSCs (**A**) The expression of miR-124, miR-137 and let-7 was analyzed in phenformin-treated GSCs by qPCR following 3 days of phenformin treatment. (**B**) GSCs were transduced with lentivirus vectors expressing a control or miR-124, miR-137 and let-7 antagomiRs. Inhibition of miR-124 and let-7 expression using specific antagomiRs abrogated the inhibitory effect of phenformin on GSC self-renewal in 10 days. * in A–B represents the statistical analyses that were performed for phenformin treated and untreated cells in each miRNA group. + represents the statistical analysis comparing anti-miRNAs + phenformin vs control + phenformin. *p < 0.05; **p < 0.01; *** and + *p* < 0.001; and ****p < 0.0001. (**C**) HMGA2 and (**D**) let-7 expression in the different subtypes of GBM was analyzed using TCGA database. (**E**) HMGA2 mRNA expression was analyzed in GSCs transduced with lentivirus vectors expressing let-7 in control or phenformin-treated cells using qPCR. (**F**) HMGA2 mRNA expression was analyzed in GSCs transduced with lentivirus vectors expressing control or let-7 antagomiRs with and without phenformin treatment using qPCR. (**G**) GSCs were transduced with a lentivirus vector expressing a HMGA2 plasmid lacking the 3′-UTR. The cells were treated with phenformin (100 μM) for 10 days and the GSC self-renewal was determined. Statistical analyses * in E–G were performed for comparing to untreated control cells (represented by the first blue columns), + for comparing to phenformin treated control cells (the red columns on the left). * or + *p* < 0.05; ** or ++ *p* < 0.01; *** or +++ *p* < 0.001; and **** or ++++ *p* < 0.0001. All the figures shown here are representative results of at least three experiments with different GSCs (listed in M&M section) that gave similar results.

Metformin has been reported to exert its anti-cancer effects via miRNAs that are associated with energy metabolic pathways or with stemness and cell cycle regulation [41] by the induction of DICER expression [27]. However, there are no reports on miRNA regulation by phenformin. Using qPCR analysis, we found that the expression of miR-124, 137 and let-7 was significantly increased following phenformin treatment (Figure 2A), whereas metformin induced a significant increase only of let-7 and miR-137 expression (Supplementary Figure S2). These data demonstrate that different miRNAs may be involved in the effects of phenformin and metformin on GSCs. In contrast, phenformin did not induce any significant changes in the expression of miR-34, miR-125b, miR-197, miR-372, miR-140 and miR-210 (data not shown).

Using specific antagomiRs we demonstrated that inhibition of miR-124 and let-7 expression decreased some of the inhibitory effect of phenformin on GSC self-renewal, whereas inhibition of miR-137 expression did not have a significant effect (Figure 2B).

Let-7 acts as a tumor suppressor miRNA and has been reported to modulate CSC self-renewal and mesenchymal transformation by targeting HMGA2 [42]. Since phenformin inhibited both the stemness and mesenchymal markers of GSCs, we further examined the effect of phenformin on HMGA2 expression and the role of let-7 in its effect. Using TCGA data analysis, we demonstrated that the expression of HMGA2 was increased in the mesenchymal GBM compared to the G-CIMP subtype that exhibits a better prognosis (Figure 2C), whereas the expression of let-7 exhibited an opposite pattern of expression (Figure 2D). Treatment of GSCs with phenformin or overexpression of let-7 induced a significant downregulation of HMGA2 mRNA levels and phenformin treatment of these cells did not result in additional decrease in HMGA2 expression (Figure 2E). Transfection of the cells with a let-7 antagomiR increased the expression of HMGA2 in the cells and abrogated the inhibitory effect of phenformin on the expression of HMGA2 (Figure 2F). We further demonstrated that transduction of GSCs with a lentivirus vector expressing HMGA2 which lacks 3′-UTR (a let-7 “resistant” HMGA2), decreased the inhibitory effect of phenformin on cell renewal of the GSCs (Figure 2G), suggesting that the targeting of HMGA2 by let-7 mediated at least some of phenformin effects.

**Figure 3:**
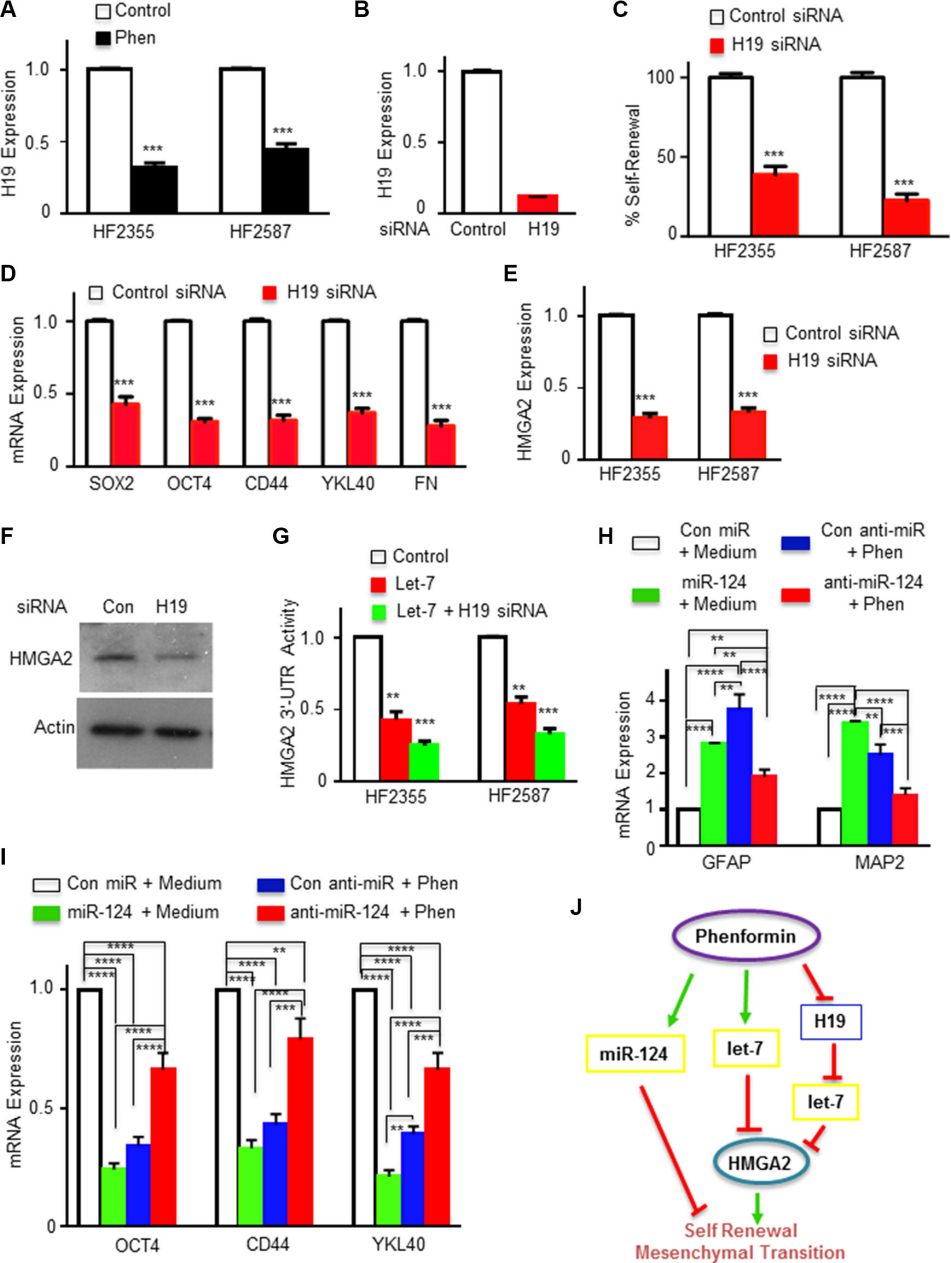
Phenformin regulates the H19/let-7/HMGA2 pathway in GSCs (**A**) H19 expression in phenformin (100 μM) treated GSCs was analyzed using qPCR. (**B**) Silencing of H19 in GSCs (HF2355) using siRNA oligonucleotides. (**C**) Silencing of H19 in GSCs significantly inhibited GSC self-renewal. (**D**) The expression of stemness and mesenchymal markers in H19-silenced GSCs (HF2355) was determined using qPCR. (**E**) HMGA2 expression in H19-silenced GSCs (HF2355 and HF2587) was analyzed using qPCR. (**F**) Western blot analysis of HMGA2 protein expression in GSCs (HF2355) confirmed that silencing H19 downregulated HMGA2 protein expression. (**G**) GSCs were transduced with a lentivirus vector expressing a HMGA2 3′-UTR plasmid tagged with luciferase. The cells then were transfected with let-7 or let-7 and H19 siRNA. Let-7 and H19 effects were analyzed by measuring luciferase activity. (**H** and **I**) mRNA expression of neuronal, stemness and mesenchymal markers was analyzed in GSCs transduced with lentivirus vectors expressing control, miR-124 or miR-124 antagomiRs with and without phenformin treatment using qPCR. (**J**) A diagram depicting the molecular mechanisms underlying phenformin effects on GSCs is presented. *p < 0.05; **p < 0.01; ***p < 0.001; and ****p < 0.0001.

Another pathway that affects the activity of let-7 is its bioavailablity and sequestration by binding to H19 [43, 44]. We therefore examined the effect of phenformin on H19 expression in GSCs. As presented in Figure 3A, treatment of the HF2355 and HF2587 with phenformin decreased the expression of H19 in these cells. We then examined the role of H19 in the stemness characteristics of the GSCs and found that silencing of H19 (Figure 3B) decreased both the self-renewal (Figure 3C) and stem cell markers of these cells (Figure 3D). In addition, silencing of H19 decreased also the expression of HMGA2 (Figure 3E, 3F) and increased the inhibitory effect of let-7 on the HMGA2 as measured using the HMGA2 3′-UTR tagged to luciferase (Figure 3G). Collectively, these results suggest that phenformin regulates both the expression and bioavailability of let-7 by upregulating its expression and downregulating H19 that acts as let-7 sponge.

In addition to the let-7 pathway, we also found that silencing of miR-124 abrogated some of phenformin effects on the expression of differentiation, stemness and mesenchymal markers (Figure 3H, 3I). These results are summarized in a diagram that depicts the effects of phenformin on the stemness of GSCs via the H19/let-7/HMGA2 [45] and the miR-124 pathway (Figure 3J).

### Phenformin inhibits the growth of GSC-derived xenograft and prolongs mouse survival

**Figure 4:**
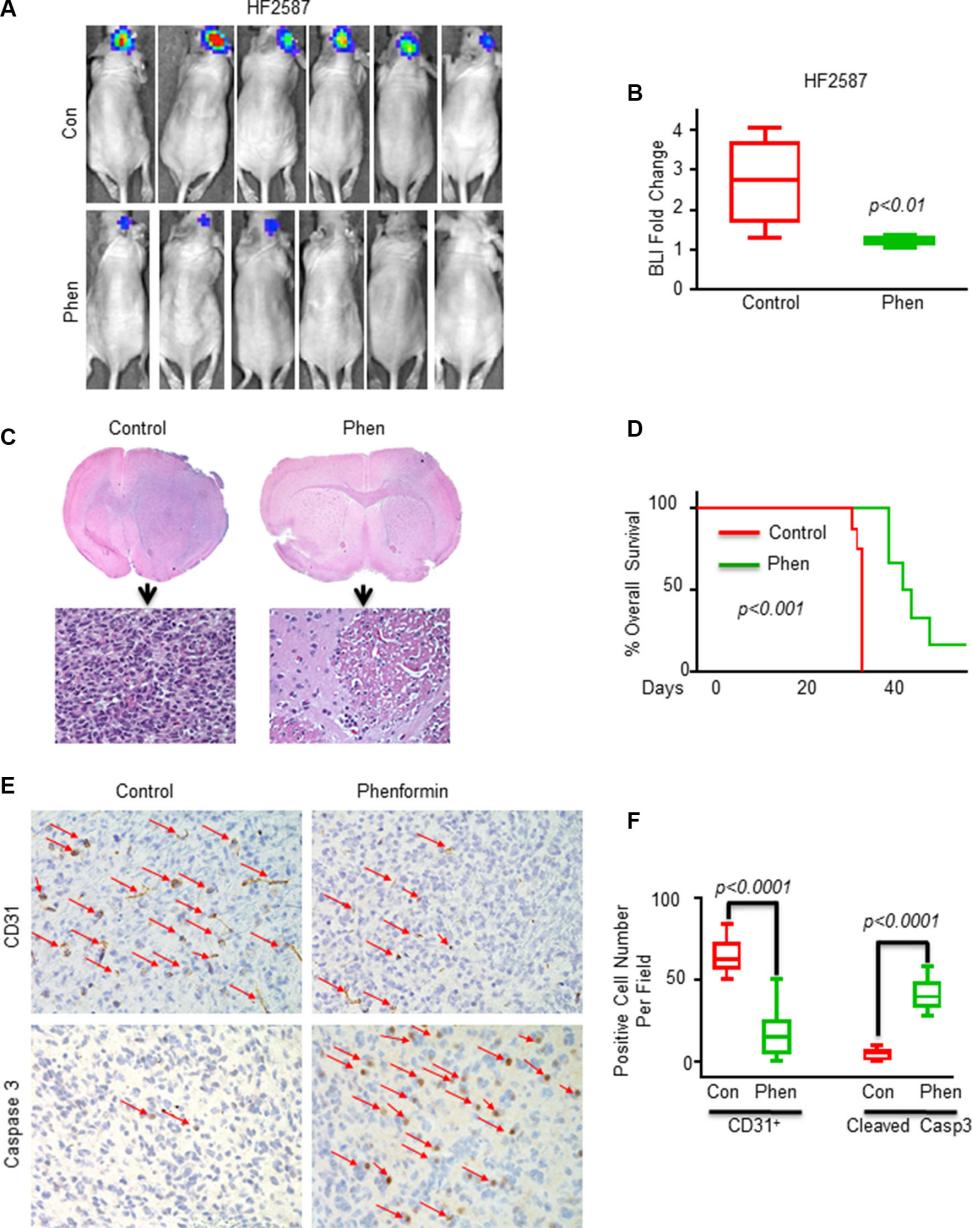
Phenformin inhibits tumor growth and angiogenesis, induces apoptosis and increases the overall survival in GSC-derived xenografts (**A**) Bioluminescence images (BLI) of control (PBS, *n* = 6) and phenformin-treated mice (1 mg/ml in drinking water, *n* = 6) after 4 weeks of phenformin treatment. (**B**) Average BLI values for the different groups were analyzed. The effect of phenformin on tumor growth was analyzed by measuring changes BLI levels before and after treatments. BLI fold change = photon flux from mice at 8 weeks after implantation of tumors (i.e. 2 weeks after treatment) divided by photon flux of mice at 2 weeks after implantation of tumors. (**C**) H&E staining of whole brain sections of control and phenformin-treated mice (*n* = 6). (**D**) Kaplan-Meier survival curves for mice treated with vehicle (*n* = 6) or phenformin (*n* = 6) were determined by both log-rank (Mantel-Cox) test and Gehan-Breslow-Wilcoxon test. (**E**) IHC staining of tumor sections of control and phenformin-treated mice (*n* = 6 in each group) was performed for both CD31 and cleaved caspase-3. Red arrows indicate positive cells for the staining of the indicated antibodies. (**F**) Quantification of CD31 and cleaved caspase-3 positive cells in control and phenformin-treated xenografts.

To analyze the effects of phenformin on GBM tumor growth and animal survival, we treated mice harboring GSC-derived xenografts with phenformin and analyzed tumor growth using *in vivo* imaging and immunohistochemical (IHC) analysis. We administered phenformin (1 mg/ml) to the mice in 5% glucose drinking water for 4 weeks and tumor growth was monitored weekly using *in vivo* luciferase imaging. Phenformin decreased tumor growth significantly as analyzed by *in vivo* imaging (Figure 4A) and after 4 weeks of treatment, the average tumor size of the phenformin-treated xenografts was about one third of the control untreated tumors (Figure 4B). Similar results were obtained when phenformin (50 mg/kg/day) was administered by intraperitoneal (i.p.) injection (Supplementary Figure S3A, S3B). These results were further validated using H&E staining (Figure 4C). In addition to its effects on tumor growth, phenformin whether administered either by drinking water, i.p. injection (data not shown) or gavage significantly prolonged the survival of the xenograft-bearing mice (Figure 4D, Supplementary Figure S3C).

To further study the mechanisms of phenformin effects *in vivo*, we analyzed the expression of CD31 and cleaved caspase-3 in tumor sections of control and phenformin-treated mice. Immunohistochemical staining showed that phenformin treatment significantly reduced CD31 expression and increased cleaved caspase-3 expression in GSC-derived xenografts (Figure 4E). The quantification of CD31 and cleaved caspase-3 expression is shown in Figure 4F. These results suggest that the therapeutic effect of phenformin in mice bearing GSC-derived xenografts is associated with the inhibition of angiogenesis and induction of tumor cell apoptosis. Therefore, the *in vivo* studies further demonstrate that phenformin has a promising potential therapeutic effect in the treatment of GBM.

The effects of phenformin on mouse survival were more pronounced than metformin's effects despite the fact that metformin concentrations were four fold higher (Supplementary Figure S3D). These results are in accordance with the *in vitro* results described in Supplementary Figure S1A.

### Phenformin enhances the inhibitory effect of TMZ on GSC growth *in vitro* and *in vivo*

**Figure 5:**
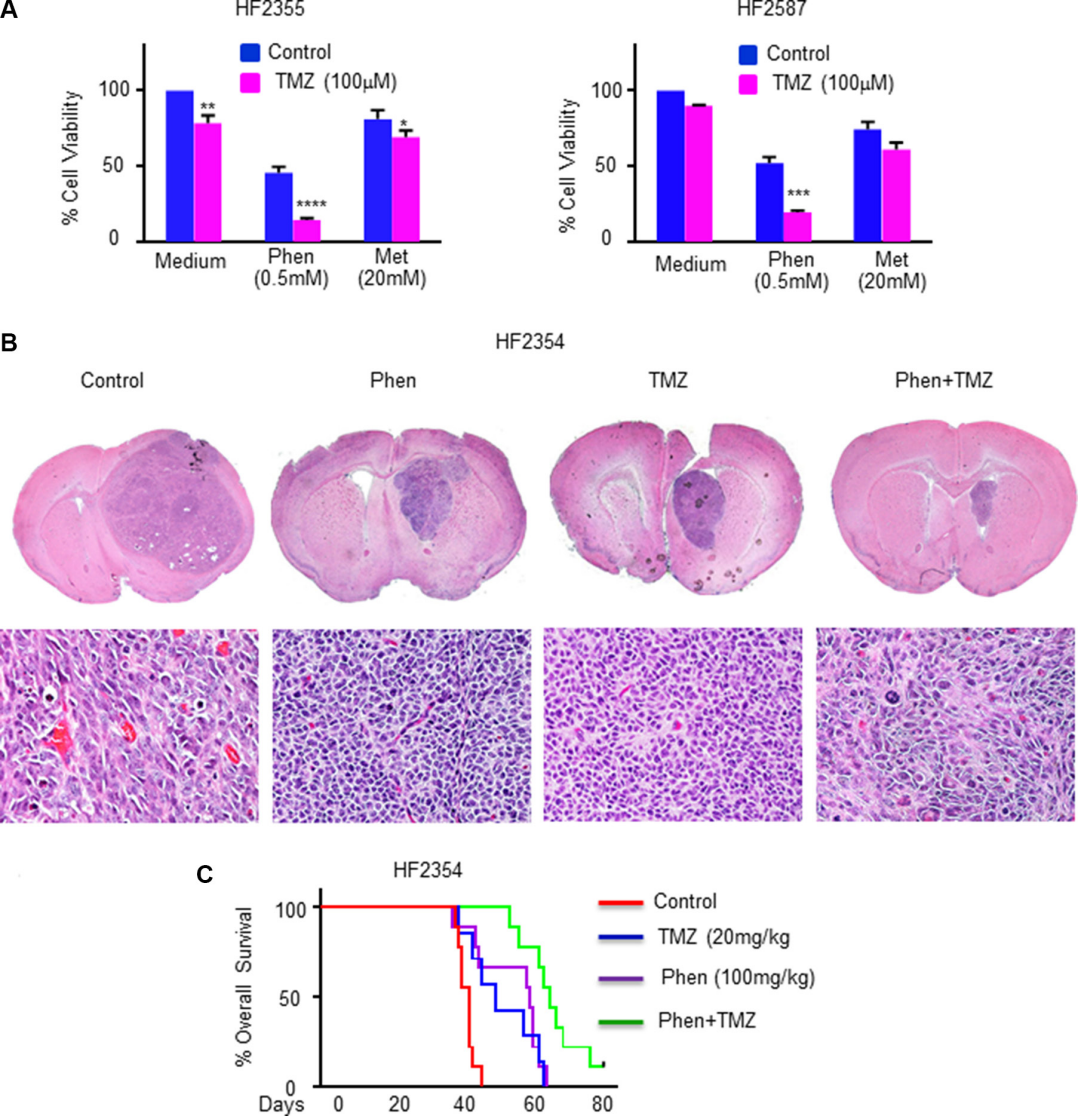
A combined treatment of phenformin and TMZ exerts an enhanced cytotoxic effect on GSCs *in vitro* and *in vivo* (**A**) MTT assay determined cytotoxicity of combined treatments of phenformin or metformin with TMZ (100 μM) for 24 hr. Statistical analysis compares TMZ treated (pink bars) vs control (blue bars). *p < 0.05, **p <0.01, ***p <0.001, ****p <0.0001. (**B**) Representative pictures of H&E staining of whole brain sections of treated mice are presented. Two weeks after implantation, GSC-derived xenograft-bearing mice were orally administered vehicle (OraPlus), phenformin (5 days/week, for 3 weeks), TMZ (5 days/week for one week) or combined treatment of phenformin and TMZ. Three mice from each group were sacrificed after 10 days post-treatment. (**C**) Kaplan-Meier survival curves for control and treated mice (*n* = 8 per group). *p* < 0.001 for all treatments compared to control mice; *p* < 0.01, for combined treatment compared to single treatments.

TMZ is the standard treatment of care for GBM patients following surgery. We found that the phenformin-induced cell death in GSCs was further increased when combined with TMZ (Figure 5A, Supplementary Figure S4). The combined effects of metformin and TMZ on GSC apoptosis were less striking than that of phenformin, and metformin even at a concentration of 40 mM exerted a smaller cytotoxic effect in TMZ-treated GSCs (Figure 5A, Supplementary Figure S4). *In vivo* studies also confirmed the enhanced effects of phenformin and TMZ on GSC-derived xenografts. Thus, combined treatment of phenformin and TMZ significantly reduced tumor growth (Figure 5B) and prolonged overall survival of xenograft-bearing mice (Figure 5C). Our results demonstrated that combined treatment with TMZ enhanced the effect of phenformin and enables the use of even lower concentrations of this drug.

### Combined phenformin and DCA treatment induces a synergistic effect on GSC death

**Figure 6:**
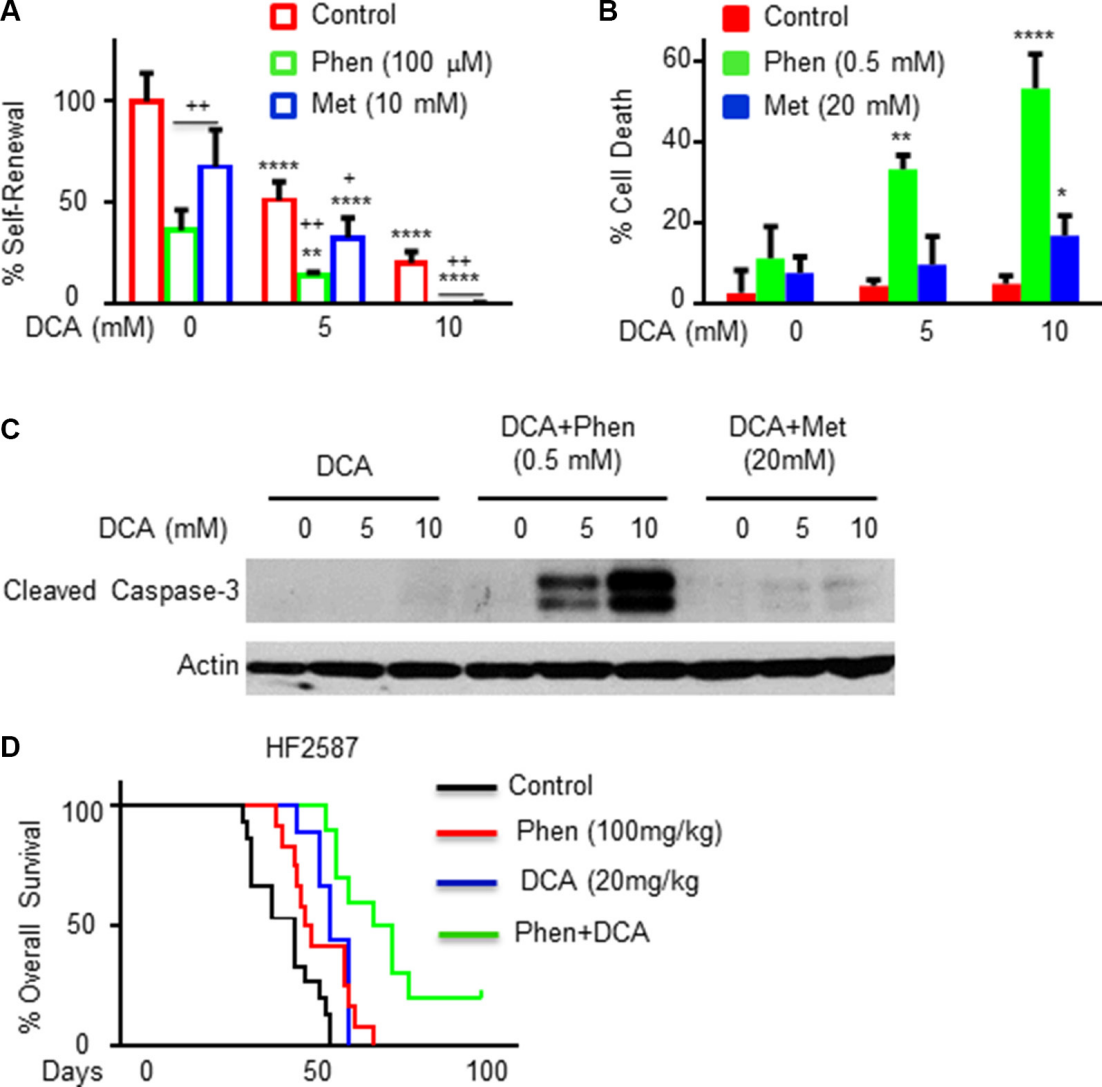
DCA treatment increases the inhibitory effect of phenformin on the stemness and survival of GSCs *in vitro* and *in vivo* (**A**) A combined treatment of phenformin or metformin with DCA on the inhibition of GSC self-renewal in 10 days. * represents the statistical analysis based on the DCA concentration, i.e, comparing 5 mM or 10 mM DCA treated groups with control untreated group ; + represents the statistical analysis that compares biguanide treatment vs. control. * or + *p* < 0.05; **p < 0.01; ***p < 0.001, **** or ++ *p* < 0.0001. (**B**) Quantification of cell death in GSCs treated with DCA in combination with phenformin or metformin for 24 hours was analyzed using the live/dead assay. *P* value was obtained by comparing 5 mM or 10 mM DCA +/− biguanide treated groups with control DCA = 0 group. *p < 0.05, **p < 0.01, ***p < 0.001, ****p < 0.0001. (**C**) Cleaved caspase-3 in the treated cells was detected by Western blot analysis. The results in A–C are representative of three different experiments/GSCs that gave similar results. (**D**) Kaplan-Meier survival curves for control (*n* = 15), phenformin (*n* = 12), DCA (*n* = 9) and combined phenformin and DCA (*n* = 10) treated mice. Two weeks after implantation, mice bearing GSC-derived xenografts were orally administered vehicle (OraPlus), phenformin (100 mg/kg, 5 days/week, for 3 weeks), DCA (20 mg/kg, 5 days/week for three week) or combined treatment of phenformin and DCA (three weeks), all brains were collected for IHC staining when mice were euthanized. *p* < 0.001 for each treatment compared to control; *p* < 0.01 for the combined compared to single treatments.

DCA is a well-established drug that has been used for the treatment of lactic acidosis, a major side effect that prompted the withdrawal of phenformin as a treatment for diabetes. DCA inhibits pyruvate dehydrogenase kinase (PDK), which suppresses lactate production and promotes cellular dependency on mitochondrial respiration [46, 47]. Recent studies demonstrated cytotoxic effects of DCA in glioma cells and a therapeutic impact in GBM patients [47]. We found that DCA decreased the self-renewal of GSCs in a dose-dependent manner and that combined treatment of DCA and phenformin (100 μM) induced a more significant effect (Figure 6A). Treatment of GSCs with DCA alone up to a concentration of 10 mM did not induce a significant degree of GSC death (Supplementary Figure S5, Figure 6B and 6C). However, a combined treatment of DCA and phenformin induced a large degree of cell apoptosis (Figures 6B and 6C, Supplementary Figure S5). Moreover, a combined treatment of phenformin and DCA exerted a more significant effect on the overall survival of mice bearing GSC-derive xenografts (Figure 6D).

A combined treatment of DCA and metformin at higher concentrations than phenformin, increased GSC death and inhibited GSC self-renewal to a much lower level (Figures 6A–6C, and Supplementary Figure S5A). We also found that treatment of the mice with phenformin or phenformin and DCA did not change lactate levels in the serum of the treated mice (Supplementary Figure S5B, S5C).

## DISCUSSION

GBM has a dismal prognosis that is partly attributed to the presence of GSCs that exhibit self-renewal abilities and resistance to radiation and chemotherapy. In this study, we explored the effects of phenformin on the proliferation, apoptosis and stemness of GSCs. Most anticancer drugs employed currently inhibit mainly tumor cell proliferation and induce cell death, but have limited efficacy in targeting cancer stem cells. Our study shows that phenformin attenuates the self-renewal and stemness of GSCs and induces cell death when administered at higher concentrations. Within a very low concentration range that is 400-fold lower than that of metformin, phenformin inhibits GSC self-renewal and stemness-related proteins. Moreover, GSCs appear to be much more sensitive than glioma cells to the effect of phenformin, further indicating that it can efficiently target GSCs.

One of the barriers to successful treatment of GBM is the eradication of the GSC subpopulation. Despite the fact, that GSCs represent only a small percentage of the tumor cells in GBM, they are resistant to current therapeutic modalities and implicated in tumor recurrence. The potent antitumor effect of phenformin on GSCs demonstrated in this study highlights the potential of this drug as a novel and potent therapeutic agent for the treatment of GBM.

Metformin has been reported to exert its anti-cancer effects through miRNA modulation via the induction of DICER expression [29]. The miRNAs induced by metformin are associated mainly with energy metabolism pathways; however, additional miRNAs involved in stemness and cell cycle regulation have been also reported to be modulated by this drug [41]. We found that phenformin induced the upregulation of miR-124, miR-137 and let-7 in the GSCs and that let-7 and miR-124 played a role in the inhibitory effect of phenformin on the self-renewal of these cells. Moreover, we found that the effect of phenformin on GSCs was mediated by the downregulation of HMGA2 via the targeting of this gene by let-7. HMGA2 acts as an oncogene in a large number of tumors and has been reported to be a direct target of let-7 [48]. Recently, HMGA2 has been also reported to mediate the inhibitory effect of let-7 on cancer stem cells in anaplastic astrocytoma [49, 50].

In addition to the upregulation of let-7 expression, we found that phenformin also increased the bioavailability of let-7 by inhibiting the expression of H19 that acts as a let-7 sponge [43]. Indeed, recent studies demonstrated that H19 acts as an oncogenic long non-coding RNA (lncRNA) [51, 52] that contains binding sites for let-7 miRNA family and modulate let-7 bioavailability [43, 53]. Our data demonstrate that silencing of H19 decreased the stemness of GSCs and enhanced the ability of let-7 to inhibit HMGA2 expression. Thus, we conclude that phenformin mediates its effects on GSC stemness via the upregulation of let-7 expression and downregulation of H19 that further increases let-7 bioavailability and ability to inhibit HMGA2 expression. The effects of let-7 on additional pathways that regulate GSC function and survival and the role of miR-124 in phenformin effects are currently being studied. In contrast to phenformin, metformin did not upregulate the expression of miR-124 in GSCs. Since this miRNA has been reported to inhibit the self-renewal of GSCs [54] and the activation of STAT3 signaling [55], the differential effect of metformin and phenformin on miR-124 expression may be associated with the increased inhibitory effect of phenformin on GSCs self-renewal and mesenchymal transition.

In addition to its inhibitory effects on GSC stemness and survival *in vitro*, phenformin also inhibited the growth of GSC-derived xenografts. The effects of phenformin on glioma xenografts *in vivo* was not yet reported, whereas, only few studies reported metformin effects on tumor growth or mouse survival [11, 56, 57]. Recently, Sato et al. demonstrated that pretreatment of glioma cells *in vitro* with metformin resulted in prolonged survival of xenograft-bearing mice [56]. Another study demonstrated the inhibitory effect of metformin on GBM tumor growth *in vivo* but did not demonstrated an effect of metformin on the survival of xenograft-bearing mice [58]. In the current study, we demonstrated that phenformin inhibited GBM tumor growth and prolonged overall survival of mice bearing GSC-derived xenografts. We also found that phenformin treatment exerted an anti-angiogenic effect as was reflected by the decreased expression of CD31 in the treated tumors and increased cell apoptosis, as indicated by the increased number of caspase-3 positive tumor cells. Indeed, recent studies have demonstrated that both metformin and phenformin inhibited angiogenesis in breast cancer and that phenformin was significantly more potent than metformin [58]. The *in vivo* apoptotic effect of phenformin is in accordance with our *in vitro* results in GSCs. Altogether, these results further emphasize the clinical potential of phenformin in GBM therapy.

Although phenformin induced a significant therapeutic impact on GSC-derived xenografts, it is unlikely to be employed as a single agent therapy for the treatment of GBM. Our results demonstrated that combined treatment of phenformin with TMZ enhanced the cytotoxic effects of phenformin and enabled the use of lower concentrations of this drug, thus increasing its efficacy and safety. Similarly, the combined treatment of phenformin with DCA resulted in enhanced cytotoxic effects that were evident with lower concentrations than each drug alone. Analyzing the therapeutic effects of the combined treatments of phenformin with TMZ or DCA demonstrated that phenformin can synergize with other GBM treatments that increase its efficacy and safety. Recent studies indicated that another way to sensitize tumor cells to phenformin is by decreasing the rate of glycolysis in these cells [59].

Metformin replaced phenformin for the treatment of diabetes mainly due to the lactic acidosis side effects of this drug. This, however, may not apply for the treatment of GBM for the following reasons. The dose of metformin for diabetes treatment is approximately 10–20 times higher than that of phenformin; however, phenformin can induce glioma cell death in concentrations 80-fold and lower and attenuate GSC self-renewal in a concentration of at least 200-fold lower than metformin. Moreover, a recent study demonstrated that treatment of mice with phenformin (100 mg/kg, o.p) for 6 weeks did not affect plasma insulin or glucose levels nor did it increase blood lactate levels [15]. Similarly, we also found that treatment of mice bearing GSC-derived xenografts with phenformin did not increase blood lactate levels.

DCA has been reported to decrease the effects of biguanides on lactate production [46] and therefore a combined treatment of DCA and phenformin is expected to inhibit lactic acidosis in response to treatment with phenformin. Our findings demonstrated that the combined treatment of phenformin and DCA resulted in synergistic cytotoxic effects that were evident with lower concentrations of each drug alone. Moreover, a combined treatment of mice bearing GSC-derived xenografts with phenformin and DCA induced a more pronounced effect on the survival of the mice compared to each treatment alone, suggesting that DCA not only can minimize the most common side effects of phenformin but also increases its anti-tumor effects. These results suggest that the risk/benefit ratio of phenformin treatment of GBM may be significantly lower than phenformin treatment of diabetes.

Collectively, our results indicate that phenformin may be a novel potent drug for the treatment of GBM and for the targeting of GSCs either alone or in combination with other GBM therapeutics.

## MATERIALS AND METHODS

### Materials

Metformin and phenformin were purchased from Sigma-Aldrich (St. Louis, MO). Anti-CD44, HMGA2, cleaved caspase-3 and PARP antibodies were purchased from Cell Signaling Technology, Inc. (Danvers, MA). Anti-actin was from Sigma-Aldrich.

### Isolation and identification of GSCs

All human materials were used in accordance with the policies of the Institutional Review Board at Henry Ford Hospital. The generation of GSCs and their characterization were recently described [37, 39, 60–62]. The GSCs that were used in this study are HF2414, HF2355, HF2354, HF2359, HF2927 and HF2587. Spheroids were maintained in neurosphere medium and examined for the expression of CD44, Bmi-1, CD133, Musashi-1, Sox2 and nestin, self-renewal, expression of astrocytic and neuronal markers upon plating on poly-D-ornithine in serum-containing medium and for their tumorigenic potential in nude mice as previously reported [36, 61].

### Western-blot analysis

Western blot analysis was performed as described [36]. Equal loading was verified using an anti-β-actin antibody.

### Real-time quantitative PCR analysis

Total RNA was isolated from cultured cells using QIAzol reagent (Qiagen, Valencia, CA) according to the manufacturer's protocol. 0.5 μg of RNA was employed to synthesize cDNA by Thermoscript (Invitrogen, Carlsbad, CA) with oligodT primers. We employed the SYBR green quantitative PCR method to analyze mRNA expression levels. For internal control we employed S12 mRNA levels. Primers for stemness and mesenchymal markers were previously reported (37.38).

For the expression of miRNAs in the different cells, total RNA was isolated from the sample of interest using miReasy total RNA isolation kit from Qiagen that isolates RNA fraction with sizes < 200 bp. 1mg of total RNA was processed according to Qiagen miScript System principle and procedures (miScript II RT kit). miRNAs forward primers were generated using sequences from miRBase and were obtained from Applied Biosystems (Foster City, CA). The reactions were run in triplicates. The relative expression of the specific miRNAs was calculated using the comparative (CT) method after normalization to U6 snRNA. Quantitative miRNA or mRNA expression data were acquired and analyzed using the ABI Prism 7000 Sequence Detection System (Applied Biosystems). Data were further analyzed by comparative CT (ΔΔCT) method, and results are expressed in arbitrary units.

### Neurosphere formation assay

The ability of GSCs to form secondary neurospheres was determined as previously described [54, 63, 64]. Briefly, disaggregated cells were subjected to the appropriate treatments, and cells were plated onto 24-well plates at a density of 10 or 100 cells/well through limiting dilutions. The number of neurospheres per well was determined 10 or 14 days thereafter for 8 different wells. Spheres that contained more than 20 cells were scored. Results are presented as percentages of maximal neurospheres formed in control untreated cells.

### *In vitro* limiting dilution assay

GSCs were plated in 96-well plates in decreasing numbers per well (50, 20, 10, 5, 2 and 1). Ten days later the generation and number of neurospheres were quantified in each well. Extreme limiting dilution analysis was performed using software available at http://bioinf.wehi.edu.au/software/elda.

### Cell growth and proliferation

GSCs were plated at a concentration of 2000 cells/well in 96 wells. Cell proliferation was determined using the ViaLight plus kit (Lonza) according to the manufacturer's guidelines.

### Gene and non-coding RNA expression in GSCs

Lentivirus vectors expressing let-7, let-7 antagomiR tagged to GFP (System Biosciences, Mountain View, CA) or HMGA2 lacking the 3′-UTR were packaged and used to infect glioma cells according to the manufacturer's protocol and as previously described [54, 65]. The transduction efficiency was more than 85% as determined by GFP expression and RT-PCR validation.

Silencing of H19 was performed using siRNA oligonucleotides obtained from ABM (Richmond, Canada).

### Cell death assays

One day before phenformin or metformin treatment, 1.5− 2.5 × 10^5^ cells were seeded in a 6-well plate. We used two methods to analyze live/dead cells. The first method was trypan blue staining of dead cells. The live and dead cell numbers were determined using a hemacytometer. Both dead and live cell numbers were counted with a hemacytometer under microscope. The second method was the live/dead cell assay (MP03224, Molecular Probes, Invitrogen). Briefly, calcein AM and EthD-1 were added to the culture medium for a final concentration of 1 μM for calcein AM and 2.5 μM for EthD-1. The live/dead cell number was then analyzed according to the manufacturer's instructions.

### GSC-derived xenografts

Following the guidelines of Henry Ford Hospital's Institutional Animal Care and Use Committee, dissociated GSCs (3 × 10^5^ cells) transduced with a lentivirus vector expressing FLuc were inoculated intracranially into nude mice (Nu/Nu) as previously described [54]. Briefly, animals were anesthetized and injected with the primary human GSCs through a 3 mm hole to the right of the bregma at a depth of 2.5 mm and a rate of 5 μl/30 seconds. Mice were treated with phenformin 2 weeks following GSC implantation according to the following protocols: Experiment 1: Vehicle (5% glucose) or phenformin (1 mg/ml in 5% glucose water) were administered in drinking water for 4 weeks. Fresh water was provided every other day and the amount of water consumed by the mice was measured daily. Experiment 2: Vehicle (OraPlus) or phenformin (100 mg/kg in OraPlus) were administered by daily oral gavage for 5 days per week for 3 weeks. Experiment 3: Saline (0.9%), or phenformin (50 or 100 mg/kg/day) were administered via intraperitoneal (i.p.) injections once daily for 5 days/week for 2 weeks. As a comparison, metformin (400 mg/kg/day) was also administrated orally for three weeks. All animals were monitored daily and sacrificed at the first signs of neurological deficit. Tumor growth in mice was monitored using Xenogen imaging system. For combined treatments, vehicle (OraPlus), phenformin (100 mg/kg in OraPlus), TMZ (20 mg/kg in OraPlus), DCA (20 mg/kg in Oraplus), phenformin (100 mg/kg) +TMZ (20 mg/kg), or phenformin (100 mg/kg) + DCA (20 mg/kg) were administered by daily oral gavage. Among the treatments phenformin and DCA were given once a day and 5 days per week for 3 weeks, and TMZ was given once a day and 5 days/week for 1 week only. The number of mice used in each experiments are listed in specific figure legends.

### Bioluminescence imaging

For *in vivo* luciferase assays, D-luciferin (150 mg/kg) was inoculated i.p. into nude mice to measure the tumor size. Bioluminescence images were obtained using the IVIS Spectrum System (Perkin-Elmer Life Sciences, Waltham, MA). Mice were imagined once a week since one week after implantation of tumors. The effect of treatment on the tumor growth was analyzed by comprising BLI change before and after treatment.

### TCGA data analysis

Level 2 processed gene expression data from the public-access clinical data tables were downloaded from the TCGA for 517 GBM. The per-sample files were merged on the Agilent probe ID to form a single data table (SAS v9.2). Expression values were assessed for each gene of interest (HMGA2, let-7) and averaged across consistent probes. Expression was averaged within gene for persons who had more than one tumor sample/aliquot analyzed. For consistency, the origin of tissue was the brain, no prior tumor was recorded and the histopathology was noted to be untreated primary (de novo) GBM.

### Statistical analysis

The results are presented as the mean values ± standard deviation. Data were analyzed using ANOVA or a Student's *t*-test with correction for data sets with unequal variances. Kaplan-Meier analysis was used to produce survival curves with differences tested between groups by the log-rank test. Data were analyzed on a log 2 scale as appropriate.

## SUPPLEMENTARY MATERIALS FIGURES


