# Supplementary material for: Repurposing phenformin for the targeting of glioma stem cells and the treatment of glioblastoma
Source: Oncotarget. 2016 Jul 29;7(35):56456–70. doi: 10.18632/oncotarget.10919 (PMC5302927; doi:10.18632/oncotarget.10919)
Supplement: Supplementary file 1 [file oncotarget-07-56456-s001.pdf]

Repurposing phenformin for the targeting of glioma stem cells and the treatment of glioblastoma

Supplementary Materials

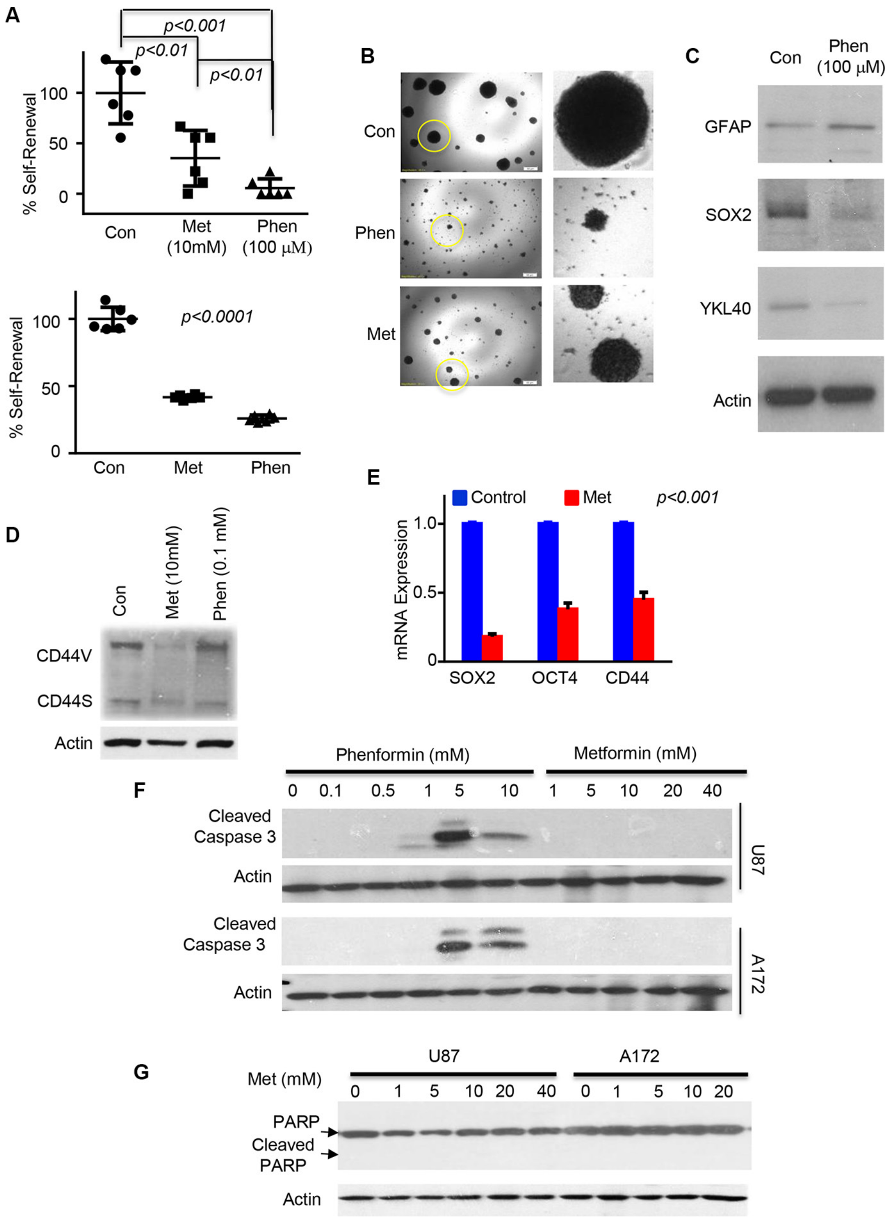

Supplementary Figure S1: Phenformin and metformin inhibit GSC self-renewal and down-regulate the expression of stemness and mesenchymal markers. (**A**) Self-renewal assay of GSCs (HF2414) that were treated with vehicle, phenformin (100 µM) or metformin (10 mM) and were plated at 10 cells/well or 100 cells/well in 24-well plates. The number of neurospheres per well was quantified after 14 days for 20 different wells. (**B**) Representative pictures of neurosphere size (HF2587) after 2 weeks of treatment with phenformine are presented. (**C**) Western Blot analysis of stemness, neuronal, and mesenchymal markers of phenformin (100 µM) treated GSCs (HF2355). (**D**) Western Blot analysis of CD44 in metformin (10 mM) or phenformin (100 µM) treated GSCs (HF2587). (**E**) GSCs (HF2355) were treated with metformin (10 mM) for 3 days and the expression of stemness markers was analyzed using qPCR. (**F**) and (**G**) Western Blot analysis of cleaved capsase 3 and cleaved PARP expression in metformin or phenformin-treated glioma cell lines.

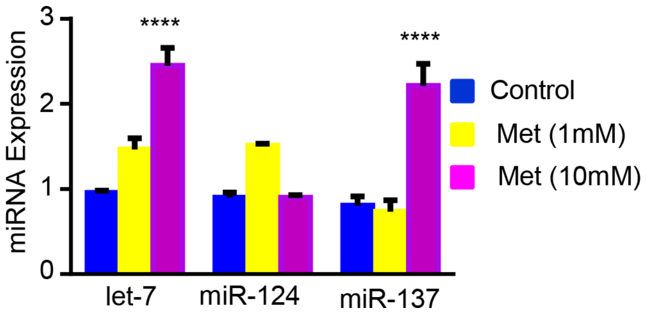

Supplementary Figure S2: Metformin regulates miRNAs expression in GSCs. GSCs were treated with control (DMEM), 1 mM or 10 mM metformin for 3 days and the expression of miR-124, miR-137 and Let-7 was determined using qPCR. The results are representative of three different experiments that gave similar results. ****$p < 0.0001$.

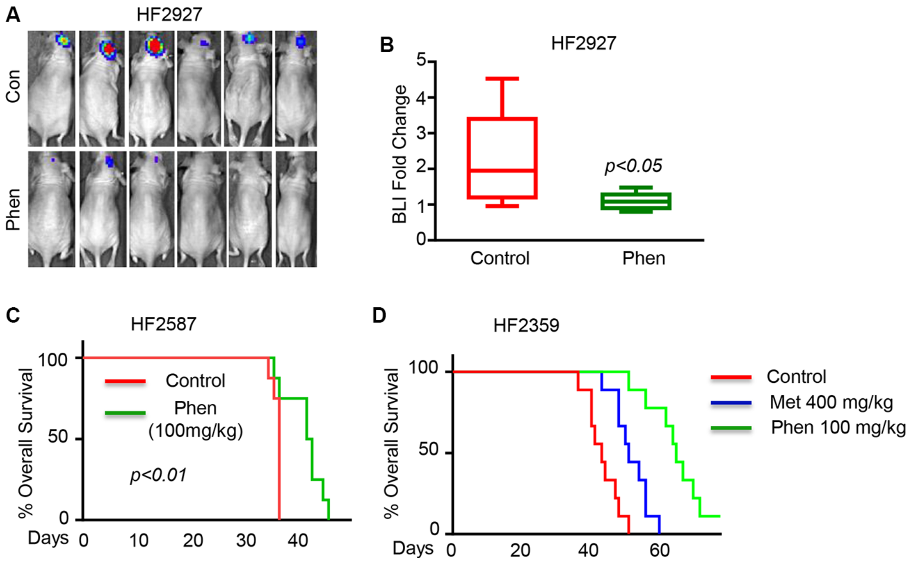

Supplementary Figure S3: Phenformin inhibits tumor growth and prolongs the survival of mice bearing GSC-derived xenografts. (**A**) Representative BLI images from control (PBS, i.p., $n = 10$) and phenformin treated mice (i.p. 50 mg/kg/day, $n = 10$). BLI was performed 2 weeks following 2 weeks of phenformin treatment. (**B**) Average BLI values for each group were analyzed, the effect of phenformin on tumor growth was analyzed by comprising BLI change before and after treatment. BLI fold change = photon flux from mice at 8 weeks after implantation of tumors (i.e. 2 weeks after treatment)/photon flux of mice at 2 weeks after implantation of tumors. (**C**) Kaplan-Meier survival curves of mice treated with vehicle (PBS) or phenformin (100 mg/kg/day) administered via oral gavage. (**D**) Kaplan-Meier survival curves of mice treated with vehicle (PBS), metformin (400 mg/kg/day) or phenformin (100 mg/kg/day) administered via oral gavage.

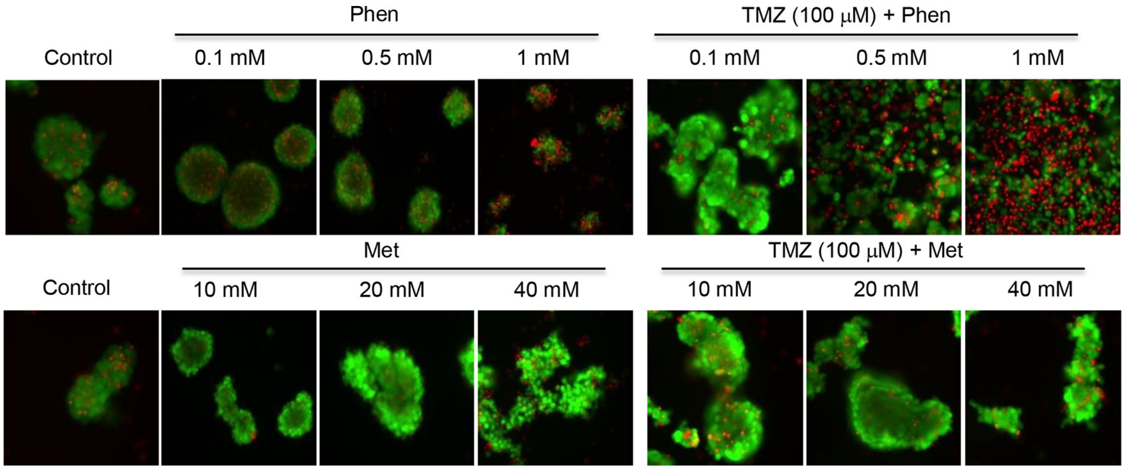

Supplementary Figure S4: A combined treatment with phenformin and TMZ exerts an enhanced effect on GSC death. Live/dead cell assay of GSCs (HF2355) that were treated with phenformin or metformin in combination with TMZ (100 μM) for 24 hours. The results are representative of three different experiments that gave similar results.

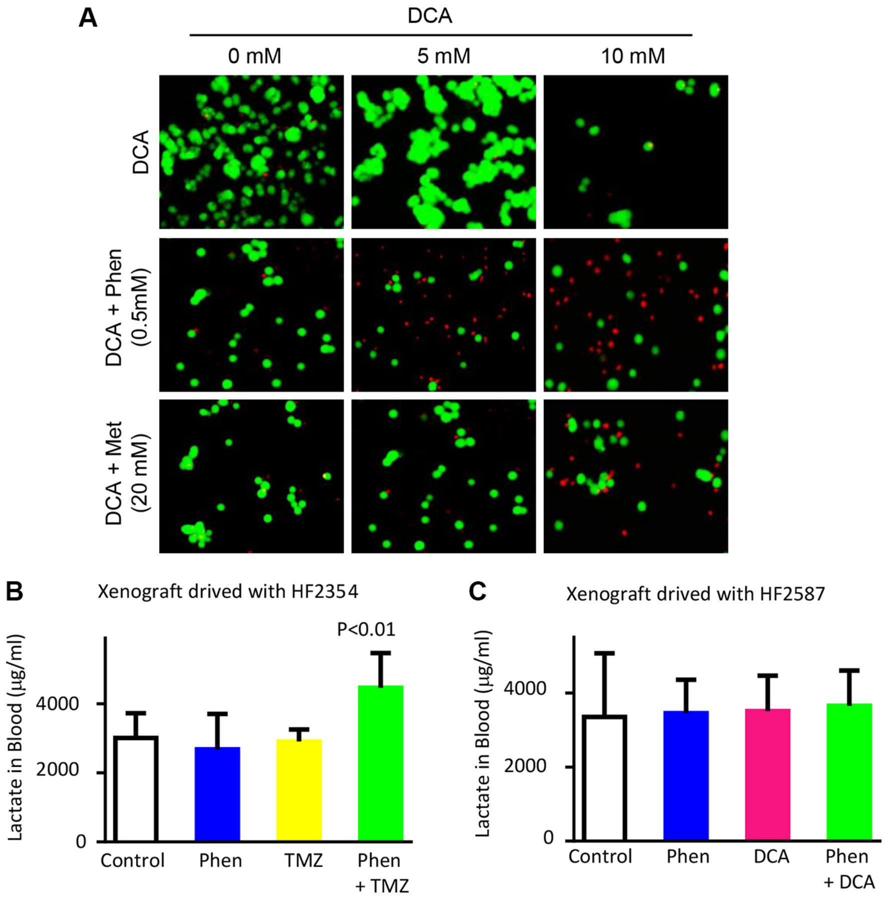

Supplementary Figure S5: DCA treatment increases the effect of phenformin on GSC death. (**A**) GSCs were treated with DCA (5 and 10 mM) in combination with 0.5 mM phenformin or 20 mM metformin for 24 hours. Cell death was analyzed using the live-dead assay. The results are representative of three different experiments that gave similar results. (**B** and **C**) Lactate level in mice bearing GSC-derived xenografts after treated with PBS, phenformin (100 mg/kg/day for 3 weeks), TMZ (20 mg/kg/day for 5 days), DCA (20 mg/kg/day for 3 weeks) or combined treatment of phenformin + TMZ or phenformin + DCA. Mice blood was collected when mice were euthanized. Number of mice in each group ≥ 7.
